# Anti-VEGF Agents with or without Antimetabolites in Trabeculectomy for Glaucoma: A Meta-Analysis

**DOI:** 10.1371/journal.pone.0088403

**Published:** 2014-02-11

**Authors:** Qi Xiong, Zhiliang Li, Zhaohui Li, Yi Zhu, Sancar Abdulhalim, Ping Wang, Xiaojun Cai

**Affiliations:** 1 Department of Ophthalmology, Zhongnan Hospital of Wuhan University, Wuhan, Hubei, P.R. China; 2 Department of Orthpedics, Union Hospital of Tongji Medical College, Huazhong University of Science and Technology, Wuhan, Hubei, P.R. China; Duke University, United States of America

## Abstract

**Purpose:**

We aimed to evaluate the intraoperative application of antimetabolites compared with anti-vascular endothelial growth factor (VEGF) agents with or without antimetabolites in trabeculectomy (Trab) for glaucoma.

**Methods:**

Relevant studies were selected through extensive search using PubMed, EMBASE, the Cochrane Library, and Web of Science databases in August 2013. The primary efficacy estimate was measured using weighted mean difference of the percentage of intraocular pressure reduction (IOPR%) from baseline to end-point, and the secondary efficacy estimates were odds ratio (OR) and 95% confidence interval (CI) for complete success rate and qualified success rate. ORs were also used to measure the tolerability estimate for adverse events. Meta-analyses of fixed or random effects models were conducted using RevMan software 5.2 to pool the results of the studies included. Heterogeneity was assessed using Chi^2^ test and the I^2^ measure.

**Results:**

Nine studies enrolling a total of 349 patients were included. The weighted mean difference of IOPR% from baseline was 7.23 (95% CI: 2.57–11.89) for antimetabolites vs. anti-VEGF agents and 3.96 (95% CI: −4.18–12.10) for antimetabolites vs. anti-VEGF agents plus antimetabolites. The pooled ORs comparing antimetabolites with anti-VEGF agents were 2.37 (95% CI: 0.78, 7.21) for the complete success rate and 1.93 (95% CI: 0.52, 7.16) for qualified success rate. The pooled ORs comparing antimetabolites with anti-VEGF agents plus antimetabolites were 1.43 (95% CI: 0.48, 4.29) for the complete success rate and 2.11 (95% CI: 0.12, 37.72) for qualified success rate. The rates of adverse events did not significantly differ between antimetabolites and anti-VEGF agents, with pooled ORs of 0.86 (0.28–2.69) for bleb leakage, 3.01 (0.45–20.10) for choroidal effusion, 0.96 (0.23–3.98) for flat anterior chamber, and 0.90 (0.12–6.60) for hypotony. Further, the rates of adverse events were similar between antimetabolites and anti-VEGF agents plus antimetabolites, with pooled ORs of 0.40 (0.08–2.00) and 8.00 (0.93–68.59) for bleb leakage and hypotony, respectively.

**Conclusions:**

In comparison with anti-VEGF agents, antimetabolites were more effective in lowering IOP in Trab, while the intraoperative application of these two types of agents did not indicate statistically significant differences in the complete success rate, qualified success rate, or incidence of adverse events.

## Introduction

Glaucoma is characterized by optic nerve atrophy and visual field defects, which is one of the many clinically common irreversible blinding eye diseases, seriously threatening the optic nerve function. There were 60.5 million people with glaucoma worldwide in 2010, and it is predicted that glaucoma will affect more than 79.6 million people by 2020 [1]. Glaucoma treatments, either pharmacologically or surgically, are directed toward reducing intraocular pressure (IOP). Since it was first introduced in 1968, trabeculectomy (Trab) has been the most effective therapy in reducing IOP in patients with medically uncontrollable glaucoma [2]–[4]. Unlike most other surgical procedures, this filtrating surgery can be successfully performed by inhibiting the wound healing process [5]. Excessive postoperative scarring of the conjunctiva and Tenon’s capsule, resulting in new water channels being blocked and poor postoperative control of IOP, has been reported to be the major reason for the failure of Trab [6], [7].

Antimetabolites, such as mitomycin C (MMC) and 5-fluorouracil (5-FU), which have been used in Trab to delay the wound healing process, can improve the success rate of surgery by inhibiting both inflammation and fibroblastic activity. Due to their nonspecific effects on cell biology, their application may lead to cell damage, followed by persistent low postoperative IOP with decreased vision, bleb leakage, corneal epithelium defect, and endophthalmitis [8], [9]. Thus, to minimize the risk of these potential adverse events, novel effective therapies involving wound healing processes, are currently undergoing experimental and clinical study.

Vascular endothelial growth factor (VEGF) is a cytokine with multiple effects on wound healing [10], [11]. In a study conducted by Li et al [12], VEGF expression was observed in aqueous humor samples of postoperative glaucoma patients and rabbits, which accelerated the proliferation of Tenon’s fibroblasts in vitro. Bevacizumab and ranibizumab, which are monoclonal antibodies against VEGF, showed promising results as a potent means to lessen scarring after filtration surgery. Several studies have demonstrated that either subconjunctival or intravitreal anti-VEGF agents may function as a potential adjuvant therapy to reduce the incidence of fibroblast proliferation and scar formation after Trab [13], [14].

Several studies have recently compared the efficacy of antimetabolites with anti-VEGF agents in inhibiting scarring after Trab. Some of these studies found antimetabolites to be more effective, while others showed anti-VEGF agents as being more effective. These inconsistent results have made it difficult to draw evidence-based conclusions that could be applied in clinical practice. To the best of our knowledge, relevant data has not yet been systematically evaluated and reported. Therefore, here we performed a meta-analysis of controlled clinical trials to assess the efficacy and tolerability of antimetabolites and anti-VEGF agents in Trab for glaucoma.

## Materials and Methods

Meta-analysis was performed according to a predetermined protocol described in the following paragraph. As outlined by the Cochrane Handbook for Systematic Reviews of Interventions [15] and PRISMA statement [16], standard systematic review techniques were followed throughout the entire process.

### Literature search

Two investigators (Q.X. and Z.L.L.) searched PubMed, EMBASE, the Cochrane Library, and Web of Science databases systematically for relevant studies in August 2013. The following search terms were used: (1) mitomycin C, or 5-fluorouracil; (2) bevacizumab, Avastin, ranibizumab, or Lucentis; and (3) trabeculectomy. A manual search was performed by checking the reference lists of the original reports and review articles in order to identify studies that were not yet included in the computerized databases. No language restriction was set.

### Inclusion and exclusion criteria

Published studies were included on the basis of the following criteria: (i) study design: controlled clinical study; (ii) population: glaucoma patients who underwent Trab or phacotrabeculectomy; (iii) intervention: intraoperative application of antimetabolites vs. anti-VEGF agents with or without antimetabolites at any concentration and dose in Trab; and (iv) outcome variables: at least one of the following: percentage of IOP reduction (IOPR%), complete success rate, and qualified success rate. Letters, reviews, duplicate publications, abstracts from conferences, unqualified control group, and full texts without raw data were excluded from this study.

### Data extraction

Two investigators (Q.X. and Z.H.L.) independently extracted data using standardized data abstraction forms. Differences were resolved by discussion with a third independent expert (X.J.C.) in ophthalmology. Information collected from these publications included the included the following: author name, publication year, study design, country or region, study duration, sample size, age and sex of the study population, IOP measurements, success rate, and incidence of adverse events.

### Assessment of study quality

The quality of clinical trials included in this study was assessed by two authors (Q.X and Z.L.L.) using a previously reported system by Downs and Blacks [17] that can assess both randomized and nonrandomized studies. The system comprises 27 items distributed among 5 subscales with regard to reporting (10 items), external validity (3 items), bias (7 items), confounding (6 items), and power (1 item). Any discrepancy in the qualitative assessment was discussed with a third investigator (Y.Z.) until a consensus was reached. The total score of each trial was expressed as a percentage of the maximum achievable score. Studies with a quality score of >50% were considered to have high quality.

### Outcome measures

The primary outcome for efficacy was IOPR%. When mean and standard deviation (SD) of IOP and IOPR were reported, we used them directly. When these were unavailable, they were calculated according to the methods described in the Cochrane Handbook for Systematic Reviews of Interventions: IOPR  =  IOP_baseline_ − IOP_end point_, SD_IOPR_  =  (SD_baseline_^2^ + SD_end point_^2^ − SD_baseline_ × SD_end point_)^1/2^. IOPR% and SD of IOPR% (SD_IOPR%_) were estimated by IOPR%  =  IOPR/IOP_baseline_ and SD_IOPR%_  =  SD_IOPR_/IOP_baseline_, respectively. For efficacy, the proportions of qualified success and complete success were used. Complete success was defined as target end point IOP without medications, and qualified success was defined as target end point IOP with or without medications. The third outcome was the incidence of adverse events, including bleb leakage, choroidal effusion, flat anterior chamber, and hypotony.

### Statistical analysis

Statistical analyses were performed using RevMan 5.2 software. We calculated pooled odds ratio (OR) for dichotomous outcomes, and weighted mean difference (WMD) or standard mean difference (SMD) for continuous outcomes. Heterogeneity among trials was assessed by inspection of graphical presentations and using Chi^2^ test and the I^2^ measure [18]. Significant heterogeneity was defined as P < 0.05 for chi-square or the I^2^ measure >50%. We used a fixed effects model to pool results when there was no significant heterogeneity; otherwise, a random effects model was used (inverse of variance method and DerSimonian and Laird method). P < 0.05 indicated statistical significance on the test for overall effect. Subgroup analysis was performed to evaluate the effect of methodological characteristics in terms of study designs, which were differentiated as retrospective (Retro), prospective (Pro) nonrandomized, and randomized.

## Results

### Literature search

**Figure 1:**
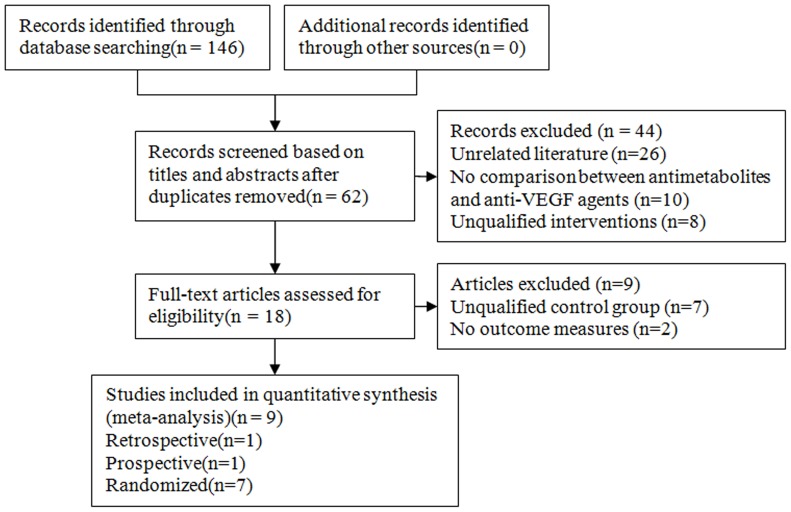
Flow chart of literature search and study selection.

In total, 146 papers were identified by our literature search. Of 146, 84 papers were duplicates; thus, these were excluded. Based on the content of the titles and abstracts of the remaining 62 papers, we excluded 44 papers for reasons outlined in Figure 1. Further 9 papers were excluded owing to unqualified control groups and lack of required outcomes. Finally, 9 eligible controlled clinical trials that met our inclusion criteria were included in this systematic review [19]–[27].

### Characteristics and quality of the included studies

Of the 9 included studies, 5 reported the use of antimetabolites vs. anti-VEGF agents, whereas the other 4 reported the use of antimetabolites vs. anti-VEGF agents with antimetabolites. The baseline characteristics of the included studies are summarized in Table 1. Seven studies were randomized controlled trials (RCTs), which were undertaken in Iran, India, Turkey, Aurora, Korea, and Australia, whereas the other 2 were prospective (Pro) nonrandomized [19] and retrospective (Retro) [27]. A total of 355 eyes of 349 patients were enrolled. The duration of follow-up ranged from 6 to 25 months. The mean ages of patients ranged from 58 to 71.7 years. With regard to quality assessment, Downs and Blacks scores of all the included studies were >16 (50%). Quality assessment details are summarized in Table 2

**Table 1:** Characteristics of included studies.

First Author(year)	Design	Location	Numbers of patients	Numbers of eyes	Mean age	Sex(male/female)		Intervention regimen	Follow-up (month)
						Antimetabolites	Anti-VEGF agents		
Jurkowska (2011)	Pro	Caucasion	62	62	71.1/70.6	11/19	16/16	5-FU/4min vs.Bevacizumab	12/12
Niflorushan (2012)	RCT	Iran	34	36	58.6/60.7	11/6	11/6	MMC/3min vs. Bevacizumab	7.8/7.4
Sengupta (2012)	RCT	India	38	38	62.1/64.1	6/4	5/5	MMC/2–3min vs. Bevacizumab	6/6
Simsek (2012)	RCT	Turkey	27	27	58/61	7/8	6/6	5-FU vs. Bevacizumab	17/17
Akkan (2012)	RCT	Turkey	42	42	64.1/64.3	11/10	13/8	MMC/ 2min vs. Bevacizumab	12/12
KAHOOK (2010)	RCT	Aurora	10	10	64.2/70.6	----	----	MMC/2min vs. Ranibizumab +MMC	6/6
Suh (2013)	RCT	Korea	36	36	60.5/64.1	21/3	10/2	5-FU/5min vs. 5-FU+ Bevacizumab	24/24
Chua (2012)	RCT	Australia	39	43	64.5/66.8	21/8	11/11	5-FU vs. 5-FU+ Bevacizumab	18/18
Freiberg (2013)	Retro	Germany	61	61	67	25/36	----	5-FU vs. 5FU+ Bevacizumab	25

RCT  =  prospective randomized controlled trial; Retro  =  retrospective; Pro  =  prospective non-randomized; 5-FU = 5-fluorouracil; MMC =  mitomycin C.

**Table 2:** Quality scoring components for 9 clinical trials included.

First Author(year)	Quality Score component					Score	
	I	II	II	IV	V	Over all	Percentage (%)
KAHOOK (2010)	11	2	4	5	3	25	78.13%
Jurkowska (2011)	10	2	4	3	1	20	62.50%
Niflorushan (2012)	11	2	4	4	3	24	75.00%
Sengupta (2012)	11	2	7	5	4	29	90.63%
Simsek (2012)	10	2	4	3	3	22	68.75%
Akkan (2012)	11	2	4	4	3	24	75.00%
Chua (2012)	11	2	4	3	1	21	65.63%
Suh (2013)	11	2	4	4	1	22	68.75%
Freiberg (2013)	9	2	4	3	1	19	59.38%

### Main results of meta-analysis

**IOPR% (antimetabolites vs. anti-VEGF agents).** Five studies involving 187 eyes compared antimetabolites with anti-VEGF agents in terms of IOPR%. Mild heterogeneity was observed between these studies (p  =  0.28, I^2^  =  21%). The combined results showed that both the agents significantly decreased IOP. Antimetabolites were found to achieve a numerically greater IOPR% from baseline, and the differences in IOPR% were statistically significant (WMD  =  7.23, 95% CI: 2.57–11.89) (Table 3). We then divided the studies into 2 subgroups according to study design (Pro nonrandomized and randomized). A statistically significant result was observed in RCTs but not in the Pro nonrandomized trial (Table 4).

**Table 3:** Percentage IOP reduction from baseline comparing antimetabolites with anti-VEGF agents with or without antimetabolites.

Trials	Antimetabolites		Anti-VEGF agents		WMD(Fixed)(95%CI)
	No.of Eyes	IOPR%[Mean(SD)]	No. of Eyes	IOPR%[Mean(SD)]	
**Antimetabolites vs. Anti-VEGF agents**					
Jurkowska (2011)	30	52.94(28.48)	32	49.83(27.78)	3.11[–10.91, 17.13]
Niflorushan (2012)	18	58.28(18.20)	18	41.64(31.51)	16.64[–0.17, 33.45]
Sengupta (2012)	10	41.94(20.54)	10	46.36(17.22)	–4.42[–21.03, 12.19]
Simsek (2012)	15	61.25(12.44)	12	49.36(8.94)	11.89[3.81, 19.97]
Akkan (2012)	21	46.93(12.83)	21	41.96(11.48)	4.97[–2.39, 12.33]
Total	94		93		7.23[2.57, 11.89]
Test for heterogeneity Ch I^2^ = 5.07, df = 4(p = 0.28); I^2^ = 21%					
Test for overall effect: z = 3.04, p = 0.002					
**Antimetabolites vs. Anti-VEGF agents + Antimetabolites**					
Kahook MY(2010)	5	55(40.3)	5	36.47(16.12)	18.53[–19.51, 56.57]
Suh W(2013)	24	54.5(18.7)	12	46.9(18.2)	7.60[–5.13, 20.33]
Freiberg FJ(2013)	34	50(22.04)	27	50(21.67)	0.00[–11.03, 11.03]
Total	63		44		3.96[–4.18, 12.10]
Test for heterogeneity Ch I^2^ = 1.37, df = 2(p = 0.50); I^2^ = 0%					
Test for overall effect: z = 0.95, p = 0.34					

CI  =  confidence interval; IOP  =  intraocular pressure; IOPR%  =  percentage intraocular pressure reduction; WMD  =  weighted mean difference.

**Table 4:** Subgroup analysis evaluating the effect of trial design on percentage IOP reduction.

subgroup	Numbers of studies		WMD(Fixed)(95%CI)	Heterogeneity			Overall effect	
				CHI^2^	P	I^2^(%)	Z	P
**Antimetabolites vs. Anti-VEGF agents**								
All trials		5	7.23[2.57, 11.89]	5.07	0.28	21%	3.04	0.002
Pro		1	3.11[–10.91, 17.13]	----	----	----	0.43	0.66
RCTs		4	7.74[2.80,12.68]	4.69	0.20	36%	3.07	0.002
**Antimetabolites vs. Anti-VEGF agents + Antimetabolites**								
All trials		3	3.96[–4.18, 12.10]	1.37	0.50	0	0.95	0.34
Retro		1	0.00[–11.03, 11.03]	----	----	----	0.00	1.00
RCT		2	8.70[–3.37,20.77]	0.29	0.59	0	1.41	0.16

RCT  =  prospective randomized controlled trial; Retro  =  retrospective; Pro  =  prospective non-randomized.

**IOPR% (antimetabolites vs. anti-VEGF agents plus antimetabolites).** Four studies involving 107 eyes compared antimetabolites with anti-VEGF agents plus antimetabolites in terms of IOPR%. No statistical heterogeneity was observed between studies (p  =  0.50, I^2^  =  0%). The combined results showed that both the agents significantly decreased IOP. The differences in IOPR% were not all statistically significant (WMD  =  3.96, 95% CI: −4.18–12.10) (Table 3). We then divided the studies into 2 subgroups according to study design (Retro and randomized). The two subgroups showed different results (Table 4).

**Complete and qualified success (antimetabolites vs. anti-VEGF agents).** All studies reported the probability of complete success, no significant difference was observed between the two groups [pooled OR  =  2.37 (95% CI: 0.78, 7.21)] (Table 5). Further, there was no significant difference between antimetabolites and anti-VEGF agents in the subgroup analyses according to study design [pooled OR  =  2.49 (0.54, 11.47) for randomized and 1.82 (0.47, 6.99) for Pro nonrandomized]. Five studies also reported the proportion of patients achieving target end point IOP with or without medications at follow-up endpoint; the difference in qualified success rate between the 2 groups was not statistically significant [pooled OR  =  1.93 (0.52, 7.16)]. For the subgroup analysis according to study design, the difference between groups was not statistically significant [pooled OR  =  2.04 (0.35, 11.91) for randomized and 1.20 (0.29, 4.98) for Pro nonrandomized] (Table 5).

**Table 5:** Complete success and qualified success comparing antimetabolites with anti-VEGF agents.

Trial	Studies(n)	Success rate, n/N(%)		OR(95%CI)	Heterogeneity			Overall effect	
		Antimetabolites	Anti-VEGF agents		CHI^2^	P	I^2^(%)	Z	P
**Complete success**									
All trials	5	71/94	56/93	2.37[0.78, 7.21]	8.03	0.09	50%	1.53	0.13
Pro	1	26/30	25/32	1.82[0.47, 6.99]	----	----	----	0.87	0.38
RCT	4	45/64	31/61	2.49[0.54,11.47]	7.70	0.05	61%	1.17	0.24
**Qualified success**									
All trials	5	79/94	67/93	1.93[0.52, 7.16]	8.97	0.06	55%	0.98	0.32
Pro	1	26/30	27/32	1.20[0.29, 4.98]	----	----	----	0.26	0.80
RCT	4	53/64	40/61	2.04[0.35,11.91]	7.73	0.05	61%	0.79	0.43

RCT  =  prospective randomized controlled trial; Pro  =  prospective non-randomized.

**Complete and qualified success (antimetabolites vs. anti-VEGF agents plus antimetabolites).** In the 2 studies that reported the probability of complete success, no significant difference was found between the two groups [pooled OR  =  1.43 (0.48, 4.29); Table 6]. There was also no significant difference between the two groups in the sensitivity analyses according to study design [pooled OR  =  1.33 (0.29, 6.06) for Retro and 1.56 (0.32, 7.60) for randomized]. Two studies also reported the proportion of patients achieving target end point IOP with or without medications at follow-up endpoint; the difference in qualified success rate between the two groups was not statistically significant [pooled OR  =  2.11 (0.12, 37.72)]. For the subgroup analysis according to design, there was no statistically significant in RCT trial [pooled OR  =  2.11 (0.12, 37.72); Table 6].

**Table 6:** Complete success and qualified success comparing antimetabolites with anti-VEGF agents combined with antimetabolites.

Trial	Studies(n)	Success rate, n/N(%)		OR(95%CI)	Heterogeneity			Overall effect	
		Antimetabolites	Antimetabolites +Anti-VEGF agents		CHI^2^	P	I^2^(%)	Z	P
**Complete success**									
All trials	2	38/48	24/32	1.43 [0.48, 4.29]	0.02	0.89	0%	0.65	0.52
Pro	1	24/28	18/22	1.33 [0.29, 6.06]	----	----	----	0.37	0.71
RCT	1	14/20	6/10	1.56 [0.32,7.60]	----	----	----	0.55	0.59
**Qualified success**									
All trials	2	53/54	36/37	2.11 [0.12, 37.72]	----	----	----	0.51	0.61
Pro	1	34/34	27/27	----	----	----	----	----	----
RCT	1	19/20	9/10	2.11 [0.12, 37.72]	----	----	----	0.51	0.61

RCT  =  prospective randomized controlled trial; Pro  =  prospective non-randomized.

### Adverse events

No significant differences in the incidence of bleb leakage, choroidal effusion, flat anterior chamber, and hypotony were found between antimetabolites and anti-VEGF agents, with the pooled ORs being 0.86 (0.28–2.69), 3.01 (0.45–20.10), 0.96 (0.23–3.98), and 0.90 (0.12–6.60), respectively (Table 7). Moreover, the rates of adverse events did not significantly differ between antimetabolites and anti-VEGF agents plus antimetabolites, with pooled ORs of 0.40 (0.08–2.00) and 8.00 (0.93–68.59) for bleb leakage and hypotony, respectively.

**Table 7:** Adverse events.

Adverse events		Studies(n)	Crude event rate, n/N		OR(95%CI)	Heterogeneity			Overall effect	
			Antimetabolites	Antimetabolites /Antimetabolites +Anti-VEGF agents		CHI^2^	P	I^2^(%)	Z	P
**Antimetabolites vs. Anti-VEGF agents**										
Bleb leak	4		5/79	6/81	0.86[0.28, 2.69]	1.14	0.77	0%	0.25	0.80
Choroidal offusion	3		3/43	0/40	3.01[0.45, 20.10]	0.01	0.99	0%	1.14	0.26
Flat anterior chamber	4		3/76	3/75	0.96[0.23,3.98]	1.00	0.80	0%	0.06	0.95
Hypatony	3		1/46	1/43	0.90[0.12, 6.60]	1.19	0.28	16%	0.11	0.92
**Antimetabolites vs. Anti-VEGF agents + Antimetabolites**										
Bleb leak	2		4/29	4/17	0.40[0.08, 2.00]	----	----	----	1.12	0.26
Hypatony	2		8/39	1/32	8.00[0.93, 68.59]	----	----	----	1.90	0.06

## Discussion

Trab is an effective surgical treatment for glaucoma. The primary factor that can lead to successful Trab is preventing exaggerated wound healing responses, which are primarily mediated by fibroblast migration and proliferation [7], [28]. Current antifibrotic drugs, such as MMC and 5-FU, can optimize surgical outcomes by avoiding conjunctival healing [29]. While these agents are associated with widespread nonselective cell death and apoptosis, resulting in severe adverse events and complications [30], [31], their application is limited and the search for an ideal pharmacological agent to modulate the wound-healing response with a safer profile is urgently needed. A multicenter study recently failed to demonstrate that subconjunctival applications of CAT-152, a humanized monoclonal antibody to TGFβ2, could prevent scar formation [32]. Several other agents, such as paclitaxel, interferon, ribozymes, p21 (WAF-1/Cip-1), and MMP inhibitors have been studied, but they have not yet been completely satisfactory [33]–[36]. VEGF, a critical component of the wound healing process, has been proved to promote angiogenesis and enhance scar formation [37]. Some studies have reported increased VEGF expression in aqueous humor samples of both human and rabbits after Trab [12]. For this reason, anti-VEGF agents may display effective action during Trab. Researchers have recently also suggested that the application of anti-VEGF agents in Trab can effectively reduce the expression of VEGF and formation of new blood vessels of the bleb, resulting in less scarring and better bleb formation, thus achieving a long-term effect of IOP control [38], [39]. There are, at present, a large number of studies comparing the effectiveness and safety of antimetabolites with anti-VEGF agents in Trab. However, there is a lack of reliable evidence-based conclusions that could be applied in clinical practice. Therefore, the present meta-analysis was undertaken to assess the clinical safety and tolerability of the aforementioned agents in Trab for glaucoma.

In the current meta-analysis, we reviewed 9 controlled clinical trials using a wide range of clinically relevant outcome measures. With regard to IOP assessment, this study found that both the agents significantly decreased IOP, but in comparison with anti-VEGF agents, antimetabolites were associated with better IOP-lowering efficacy, leading to a numerically higher percentage of IOP reduction. For the outcomes of Trab in combination with anti-VEGF agents plus antimetabolites vs. with antimetabolites alone, the differences in IOPR% were not all statistically significant. One possible reason for the finding may be that antimetabolites have widespread nonselective cell death and apoptosis effects in comparison with anti-VEGF agents. Antimetabolites not only inhibit fibroblast proliferation of the sclera and conjunctiva but also, via different pharmacological mechanisms, result in a better IOP control [23]. No significant difference was found in complete and qualified success rates between the two groups. This may be a potential reason for the different definitions of complete and qualified success. For example, complete success of Trab was defined as a 30% decline in baseline IOP in some studies [19], [24], and 20% in others studies [20], [21], [27]. Another possible reason is the small sample size and short duration of follow-up, making it difficult to draw stable and credible conclusions. There was a similar result with regard to in adverse events of the two types of agents. This may also be due to a small sample size and short duration of follow-up. As is known, the objective of antimetabolites and anti-VEGF agents as an adjuvant to Trab is to not only lower IOP but also to promote an improved and less scarred bleb formation. Anti-VEGF agents can reduce vascularization in the bleb and make it bleb more durable, thereby increasing the success rate of the surgery [24]. Here, in the included studies, characteristics and morphological features of the bleb were not evaluated. Therefore, our conclusions should be interpreted with caution.

Our study had a number of strengths. First, we strictly followed the Cochrane Handbook for Systematic Reviews of Interventions and PRISMA statement, including the literature search, data extraction, quality assessment, and statistical analysis. This makes our conclusions more scientific and reliable. Second, the subgroup analysis was performed according to study design, indicating that the conclusions from this analysis are robust.

There were also several limitations: (1) Randomization: Among the 9 included studies, 7 were RCTs, 1 was Pro nonrandomized, and 1 was Retro. Only 4 of the 7 studies provided sufficient information on how to specifically implement RCTs and described the implementation of allocation concealment. Inappropriate methods of RCTs may result in potential selection bias in the conclusion. (2) Masking: Only 1 of the 7 studies performed masking, while others did not mention masking, which may result in implementation and measurement bias in the conclusion. (3) Placebo controlled: Trials should be ideally devised as placebo-controlled studies; however, none of the trials were designed to be placebo or sham controlled, which may exaggerate the treatment effect in Trab and result in bias. (4) Publication bias: We not only performed electronic searches but also conducted a manual search to identify all potential relevant papers, including published and non-published ones, to avoid publication bias. Unfortunately, we may have failed to include some papers, particularly those published in languages other than Chinese or English. In addition, the test for publication bias was not conducted due to a limited number of studies included in our meta-analysis. (5) Heterogeneity: There was significant heterogeneity in some studies, which may reflect differences in age, gender, sample size, differences in definition of complete and qualified success, and outcome of measurements. A random effects model was used when statistically significant heterogeneity was met. (6) Follow-up: The follow-up duration in two studies was only 6 months, which may affect the long-term results of our study.

In conclusion, this is the first meta-analysis specifically answering the question of whether anti-VEGF agents are more effective and safer than antimetabolites in Trab for glaucoma. The results of this meta-analysis suggest that antimetabolites are more effective in lowering IOP in Trab in comparison with anti-VEGF agents alone; however, antimetabolites are comparable with anti-VEGF agents with regard to qualified success rate, complete success rate, and incidence of adverse events. Although there were some limitations, we believe that the results of this meta-analysis possess sufficient credibility and are worth consideration in future clinical practice. We believe that more RCTs with larger sample sizes and systematic studies are required for further confirmation of the presented results.

## Supporting Information

Checklist S1 (DOC)
